# Clinical Evaluation of the iCubate iC-GPC Assay for Detection of Gram-Positive Bacteria and Resistance Markers from Positive Blood Cultures

**DOI:** 10.1128/JCM.00485-18

**Published:** 2018-08-27

**Authors:** Paul A. Granato, Melissa M. Unz, Raymond H. Widen, Suzane Silbert, Stephen Young, Kathryn L. Heflin, Matt S. Conover, Blake W. Buchan, Nathan A. Ledeboer

**Affiliations:** a Laboratory Alliance of Central New York, Syracuse, New York, USA; b Tampa General Hospital, Tampa, Florida, USA; c TriCore Laboratories, Albuquerque, New Mexico, USA; d iCubate, Inc., Huntsville, Alabama, USA; e Medical College of Wisconsin, Milwaukee, Wisconsin, USA; Cleveland Clinic

**Keywords:** blood culture, Gram-positive cocci, iCubate, resistance genes

## Abstract

The iC-GPC Assay (iCubate, Huntsville, AL) is a qualitative multiplex test for the detection of five of the most common Gram-positive bacteria (Staphylococcus aureus, Staphylococcus epidermidis, Streptococcus pneumoniae, Enterococcus faecalis, and Enterococcus faecium) responsible for bacterial bloodstream infections, performed directly from positive blood cultures. The assay also detects the presence of the *mecA*, *vanA*, and *vanB* resistance determinants. This study comparatively evaluated the performance of the iC-GPC Assay against the Verigene Gram-positive blood culture (BC-GP) assay (Luminex Corp., Austin, TX) for 1,134 patient blood culture specimens positive for Gram-positive cocci. The iC-GPC Assay had an overall percent agreement with the BC-GP assay of 95.5%. Discordant specimens were further analyzed by PCR and a bidirectional sequencing method. The results indicate that the iC-GPC Assay together with the iCubate system is an accurate and reliable tool for the detection of the five most common Gram-positive bacteria and their resistance markers responsible for bloodstream infections.

## Abstract (precis)

The iC-GPC Assay (iCubate, Huntsville, AL) is a qualitative multiplex test for the detection of five of the most common Gram-positive bacteria (Staphylococcus aureus, Staphylococcus epidermidis, Streptococcus pneumoniae, Enterococcus faecalis, and Enterococcus faecium) responsible for bacterial bloodstream infections, performed directly from positive blood cultures. The assay also detects the presence of the *mecA*, *vanA*, and *vanB* resistance determinants.

## INTRODUCTION

Bacterial sepsis and its associated complications are a major cause of morbidity and the 10th leading cause of mortality in the United States (1). Early diagnosis and the institution of appropriate antimicrobial therapy are vital for favorable patient outcomes. Unfortunately, traditional culture and antimicrobial susceptibility test methods are time-dependent processes that often require 2 to 4 days before final test results are available.

Within recent years, a variety of methods have been developed for the rapid identification of bacterial pathogens in positive blood culture broths. These methods include peptide nucleic acid fluorescence *in situ* hybridization (PNA-FISH), matrix-assisted laser desorption ionization–time of flight mass spectrometry (MALDI-TOF MS), real-time PCR (RT-PCR) assays, and microarray-based tests (2–11). The availability of these methods to reliably identify a specific bacterial agent as the cause of bloodstream infection (BSI) within one to several hours of culture positivity has resulted in significant reductions in the time to appropriate antimicrobial therapy, length of hospital stays, mortality rates, and cost of care (7, 8, 12–14). An additional benefit from the use of some of these assays is that they concomitantly detect the presence of important resistance gene markers for methicillin resistance (*mecA*), vancomycin resistance (*vanA* or *vanB*), extended-spectrum beta-lactamases, and carbapenemases (12, 13, 15–17).

The iC-GPC Assay (iCubate, Huntsville, AL) is a molecular target amplification assay capable of detecting and identifying Staphylococcus aureus, Staphylococcus epidermidis, Streptococcus pneumoniae, Enterococcus faecalis, and Enterococcus faecium, as well as three gene resistance determinants, *mecA*, *vanA*, and *vanB*, directly from positive blood cultures. The purpose of this study was to perform a multisite comparative evaluation of the iC-GPC Assay with the Verigene Gram-positive blood culture (BC-GP) assay (Luminex Corp., Austin, TX) as the reference standard. Discordant results were arbitrated by an alternative PCR method and bidirectional sequencing.

## MATERIALS AND METHODS

### Clinical specimen collection.

Four clinical laboratories participated in this study, representing different geographic areas in the United States (Laboratory Alliance of Central New York, Syracuse, NY; Medical College of Wisconsin, Milwaukee, WI; TriCore Reference Laboratories, Albuquerque, NM; and Tampa General Hospital, Tampa, FL). Clinical specimens were collected and tested from January 2015 until October 2016. Three different automated blood culture systems were evaluated (BacT/Alert, bioMérieux, Durham, NC; Bactec, Becton-Dickinson, Cockeysville, MD; and VersaTREK, Thermo Fisher, Waltham, MA). iC-GPC and BC-GP testing was performed from blood culture bottles types that included BD Bactec Standard Aerobic/Anaerobic, VersaTREK Redox 1/2, BacT/Alert SA Standard Aerobic, and BacT/Alert FA FAN aerobic bottles.

A total of 1,134 blood cultures confirmed to contain Gram-positive cocci by Gram stain were initially enrolled in this study. A total of 53 specimens were excluded from the study due to repeat iC-GPC Assay error (18), repeat Verigene BC-GP error (5), a mixed Gram stain result (19), or incomplete specimen data (6). Of the 1,081 remaining blood cultures evaluated in this study, 879 cultures were fresh prospective positive blood cultures tested within 8 h of culture positivity, 34 were frozen prospective positive blood cultures, and 168 were frozen simulated blood cultures representing low-prevalence bacteria possessing uncommon resistance markers. Specimens were stable for up to 8 h after initial bottle positivity, and bottles were stored on the blood culture bottle system, at room temperature, or refrigerated. After 8 h, aliquots were frozen for future repeat testing or discrepant analysis. Verigene BC-GP testing was performed at two clinical sites. Sites that performed internal Verigene testing tested positive blood culture specimens on the BC-GP assay within 8 h of bottle positivity. Sites that did not perform internal Verigene testing prepared frozen aliquots of the positive blood cultures and shipped the specimens on dry ice to an external site for testing. All patient specimens were deidentified prior to enrollment and tested in accordance with protocols approved by the Pearl institutional review board (IRB).

An initial invalid iC-GPC Assay result was observed for 83/1,098 (7.6%) of the specimens tested. The most common reasons for an invalid test result were a positive-controls check failure (failure of the internal process control to complete all stages of processing) or an array registration error (failure of the iC-System to properly orient the microarray). Possible reasons for positive-control check failures include mechanical failures, high levels of PCR inhibitors in the specimen, or other fluid transfer errors within the cassette. Upon repeat testing, 66 specimens produced a valid result, decreasing the iC-GPC failure rate to 1.6% (17/1,098). Specimens with reproducible invalid results were withdrawn from the study.

A total of 302 specimens were negative for iC-GPC Assay target organisms. Of these, 141 specimens (47%) were identified as a Staphylococcus species, 102 specimens (34%) were identified as a Streptococcus species, 2 specimens were identified as a mixed culture (<0.01%), and 57 specimens (19%) could not be identified by the reference assay.

### iC-GPC Assay testing.

The iCubate system consists of an automated processor (iC-Processor), reader (iC-Reader), and a single-use closed-system test cassette that contains all reagents necessary for cell lysis, nucleic acid extraction, target amplification, and amplicon hybridization to an array of immobilized capture probes. Each immobilized capture probe has a unique nucleic acid sequence designed to hybridize to the target. A second fluorescent-labeled gene-specific detection probe contained within the closed cassette was used to detect the target after capture.

Briefly, the iC-GPC Assay was performed by transferring an aliquot of the blood culture containing Gram-positive cocci, as confirmed by Gram stain into the test cassette. Based on the different compositions of the blood culture bottle medium types evaluated, 10 μl of specimen was loaded for Bactec and VersaTREK medium types, and 3 μl of specimen was loaded for BacT/Alert medium types. After sample loading, the iC-GPC Cassette was placed in the iC-Processor. Following the completion of the extraction, amplification, and hybridization reactions, the cassette was ejected by the iC-Processor and manually transferred to the iC-Reader to detect the hybridized captured targets. The entire iC-GPC Assay was completed in approximately 4.5 h. The Verigene BC-GP assay was performed in accordance with the manufacturer's instructions.

### Preparation of simulated specimens.

Simulated specimens were prepared for low-prevalence bacteria possessing uncommon resistance markers, including S. pneumoniae and *vanA* or *vanB* negative and positive E. faecalis and E. faecium specimens. A combination of purchased strains (American Type Culture Collection [ATCC], National Collection of Type Cultures [NCTC], and Zeptometrix Corporation) and clinical strains (Washington University in St. Louis) were evaluated. Vancomycin-resistant E. faecalis and E. faecium strains were confirmed as having *vanA* or *vanB* resistance determinants by PCR and bidirectional sequencing. A total of 30 unique strains were used to prepare 168 contrived specimens. Fresh subcultures grown on tryptic soy agar (TSA)–5% sheep blood agar were prepared at 0.5 McFarland concentrations using a Sensititre nephelometer and diluted to 5 to 30 CFU/ml in saline. These samples were then inoculated into BD Bactec Plus Aerobic/F blood culture bottles with 10 ml human blood added. The inoculated bottles were incubated on the Bactec 9050 system until positivity. Frozen aliquots were provided randomized and blinded to the clinical sites.

### PCR/bidirectional sequencing assays.

PCR and bidirectional sequencing assays were used for resistance marker confirmation and for arbitration of discrepant results between the iC-GPC Assay and the Verigene BC-GP assay. PCR and bidirectional sequencing assays were developed and validated by the LabCorp Perimeter Park Genomics Facility (Morrisville, NC). Briefly, PCR primers with incorporated M13 priming sites were designed to amplify unique regions of iC-GPC Assay target organisms. Frozen aliquots of clinical specimens were used to prepare fresh subcultures. Nucleic acid extraction was performed from pure isolates using a validated NucliSENS easyMAG procedure. The extracted DNA was tested using the specified PCR assay, and amplification was confirmed by analysis on an Agarose 1000 gel. Purified PCR products were sequenced with M13 universal sequencing primers using the BigDye Terminator cycle sequencing kit to achieve bidirectional coverage. Raw data were analyzed using Sequence Analysis Software. The assays were designed to generate sequencing amplicons of ≥200 bp with trace scores of >20 and to yield consensus sequences that aligned to the target sequences when analyzed using the NCBI Basic Local Alignment Search Tool (BLAST).

## RESULTS

Table 1 shows the comparative performances of the iC-GPC and Verigene BC-GP assays as well as the resolution of the discordant results after arbitration testing. Overall, there was very good positive and negative agreement for each of the bacterial targets and antibiotic resistance determinants evaluated. For S. aureus, the overall positive and negative percent agreements were 97.0% and 99.8%, respectively, in which the iC-GPC Assay had eight false-negative and two false-positive results compared to the Verigene BC-GP assay. After arbitration testing of these discordant results, one of eight iC-GPC false-negative results was confirmed as a true negative for S. aureus, and one of two false-positive results was confirmed as a true positive for S. aureus.

**TABLE 1:** Comparative performance of iC-GPC Assay with Verigene method and resolution of discordant test results following PCR/sequencing arbitration testing

Assay target (*n* = 1,081)	Sample type	% agreement, no. of samples/total no. (95% CI [%])^*a*^		Resolution of iC-GPC Assay discordant results after arbitration testing (no./total no.)^*a*^^,^^*b*^			
		Positive	Negative	FN	FP	TN	TP
Bacterial identification							
S. aureus	Overall	97.0, 258/266 (94.2–98.5)	99.8, 813/815 (99.1–99.9)	8	2	1/8	1/2^*c*^
	Prospective	97.0, 258/266 (94.2–98.5)	99.7, 645/647 (98.9–99.9)	8	2	1/8	1/2^*c*^
	Simulated	NA, 0/0 (NA)	100, 168/168 (97.8–100)	0	0	NA	NA
S. epidermidis	Overall	98.3, 231/235 (95.7–99.3)	98.3, 832/846 (97.2–99.0)	4	14	1/4	9/14
	Prospective	98.3, 231/235 (95.7–99.3)	97.9, 664/678 (96.6–98.8)	4	14	1/4	9/14
	Simulated	NA, 0/0 (NA)	100, 168/168 (97.8–100)	0	0	NA	NA
S. pneumoniae	Overall	93.7, 59/63 (84.8–97.5)	99.9, 1,017/1,018 (99.5–100)	4	1	4/4	1/1
	Prospective	85.2, 23/27 (67.5–94.1)	99.9, 885/886 (99.4–100)	4	1	4/4	1/1
	Simulated	100, 36/36 (90.4–100)	100, 132/132 (97.2–100)	0	0	NA	NA
E. faecalis	Overall	98.7, 149/151 (95.3–99.6)	99.9, 929/930 (99.4–100)	2	1	1/2	0/1
	Prospective	96.7, 59/61 (88.8–99.1)	99.9, 851/852 (99.4–100)	2	1	1/2	0/1
	Simulated	100, 90/90 (95.9–100)	100, 78/78 (95.3–100)	0	0	NA	NA
E. faecium	Overall	98.6, 70/71 (92.4–99.8)	99.8, 1,008/1,010 (99.3–100)	1	2	0/1	1/2
	Prospective	96.6, 28/29 (82.8–99.4)	99.8, 882/884 (99.2–99.9)	1	2	0/1	1/2
	Simulated	100, 42/42 (91.6–100)	100, 126/126 (97.0–100)	0	0	NA	NA
Resistance determinant							
*mecA*	Overall	96.1, 274/285 (93.2–97.8)	95.2, 216/227 (91.5–97.3)	11	11	1/11	8/11
	Prospective	96.1, 274/285 (93.2–97.8)	95.2, 216/227 (91.5–97.3)	11	11	1/11	8/11
	Simulated	NA, 0/0 (NA)	NA, 0/0 (NA)	0	0	NA	NA
*vanA*	Overall	98.3, 59/60 (91.1–99.7)	96.9, 158/163 (93.0–98.7)	1	5	0/1	2/5
	Prospective	95, 19/20 (76.4–99.1)	93.0, 66/71 (84.6–97.0)	1	5	0/1	2/5
	Simulated	100, 40/40 (91.2–100)	100, 92/92 (96.0–100)	0	0	NA	NA
*vanB*	Overall	100, 58/58 (93.8–100)	99.4, 163/164 (96.6–100)	0	1	NA	1/1
	Prospective	100, 2/2 (34.2–100)	98.8, 87/88 (93.8–99.8)	0	1	NA	1/1
	Simulated	100, 56/56 (93.6–100)	100, 76/76 (95.2–100)	0	0	NA	NA

a NA, not applicable; 95% CI, 95% confidence interval.

b FN, false negative; FP, false positive; TN, true negative; TP, true positive.

c One specimen not available for arbitration testing.

For S. epidermidis, both the positive and negative percent agreement between the two assays was 98.3%. The iC-GPC Assay had 4 false-negative and 14 false-positive results compared to the Verigene assay. After arbitration testing, one of four false-negative results was confirmed as a true negative for S. epidermidis, while 9 of the 14 false positives were confirmed as true positives for S. epidermidis.

The results for S. pneumoniae produced a comparative positive percent agreement of 93.7% and a negative percent agreement of 99.9%. All four of the false-negative iC-GPC Assay results were confirmed as true negatives for S. pneumoniae, and the one false-positive result was confirmed as a true positive for S. pneumoniae by PCR and bidirectional sequencing resolution testing. Due to a positive percent agreement of less than 95% and because of the small prospective sample size, all discordant specimens were further characterized by traditional culture-based methods (MALDI-TOF MS). MALDI-TOF MS confirmed the iC-GPC Assay result for all five discordant samples.

For the enterococci, E. faecalis had 98.7% positive and a 99.9% negative overall agreement between the iC-GPC Assay and Verigene assay. Of the two false-negative results, one was confirmed as a true negative for E. faecalis, while the one false positive was confirmed as a true false positive for E. faecalis. For E. faecium, the positive percent agreement was 98.6%, and the negative percent agreement was 99.8%. The one false-negative result was confirmed as a true false negative for E. faecium, while one of the two false-positive results was confirmed as a true positive for E. faecium.

For the detection of the antibiotic resistance determinants, the *mecA* gene had 96.1% positive agreement and 95.2% negative agreement between the two assays. Of the 274 observed true-positive *mecA* results, 269/274 (98.2%) were confirmed as *mecA* positive by PCR and bidirectional sequencing. The iC-GPC Assay had 11 false-negative and 11 false-positive results compared to the Verigene assay. After arbitration testing, 1 of the 11 false-negative results was confirmed as a true negative for *mecA*, while 8 of the 11 false-positive results were confirmed as true positives for *mecA*.

For the *vanA* determinant, the positive and negative agreements between the two assays were 98.3% and 96.9%, respectively. Of the 19 observed true-positive *vanA* results, 19/19 (100%) were confirmed as *vanA* positive by PCR and bidirectional sequencing. The one false-negative result was confirmed as a true false negative for *vanA*, while two of the five false positives were confirmed as true positives for *vanA*. The positive and negative agreements for the *vanB* resistance determinant were 100% and 99.4%, respectively. Of the 2 observed true-positive *vanB* results, 2/2 (100%) were confirmed as *vanB* positive by PCR and bidirectional sequencing. The one false-positive iC-GPC Assay result was confirmed as a true positive for *vanB* following arbitration testing.

The iC-GPC Assay was also evaluated across three of the most common types of blood culture bottle systems, BacT/Alert, Bactec, and VersaTREK. iC-GPC Assay target performance by blood culture bottle type is presented in Table 2. The overall percent agreements for prospective specimens collected in the various medium types are as follows: BacT/Alert medium, 95.5% (378/396); Bactec medium, 94.4% (234/248); and VersaTREK medium, 93.7% (252/269). When the performance of the iC-GPC Assay was compared against the BC-GP assay, there was no statistical difference observed between the study sites and the types of blood culture bottles used.

**TABLE 2:** Comparative performances of prospective samples by blood culture bottle type

Assay target (*n* = 913)	Bottle type	% agreement, no. of samples/total no. (95% CI [%])^*a*^		No. of results^*b*^	
		Positive	Negative	FN	FP
S. aureus	BacT/Alert	99.1, 116/117 (95.3–99.9)	100, 279/279 (98.6–100)	1	0
	Bactec	95.7, 88/92 (89.4–98.3)	99.4, 155/156 (96.5–99.9)	4	1
	VersaTREK	94.7, 54/57 (85.6–98.2)	99.5, 211/212 (97.4–99.9)	3	1
S. epidermidis	BacT/Alert	98.8, 83/84 (93.6–99.8)	98.1, 306/312 (95.9–99.1)	1	6
	Bactec	95.5, 63/66 (87.5–98.4)	98.9, 180/182 (96.1–99.7)	3	2
	VersaTREK	100, 85/85 (95.7–100)	96.7, 178/184 (93.1–98.5)	0	6
S. pneumoniae	BacT/Alert	92.3, 12/13 (66.7–98.6)	100, 383/383 (99.0–100)	1	0
	Bactec	85.7, 6/7 (48.7–97.4)	99.6, 240/241 (97.7–99.9)	1	1
	VersaTREK	71.4, 5/7 (35.9–91.8)	100, 262/262 (98.5–100)	2	0
E. faecalis	BacT/Alert	96.6, 28/29 (82.8–99.4)	99.7, 366/367 (98.5–100)	1	1
	Bactec	83.3, 5/6 (43.7–97.0)	100, 242/242 (98.4–100)	1	0
	VersaTREK	100, 26/26 (87.1–100)	100, 243/243 (98.4–100)	0	0
E. faecium	BacT/Alert	100, 6/6 (61.0–100)	99.7, 389/390 (98.6–100)	0	1
	Bactec	100, 1/1 (20.7–100)	100, 247/247 (98.5–100)	0	0
	VersaTREK	95.4, 21/22 (78.2–99.2)	99.6, 246/247 (97.7–99.9)	1	1
*mecA*	BacT/Alert	99.1, 112/113 (95.2–99.8)	91.4, 85/93 (83.9–95.6)	1	8
	Bactec	92.6, 75/81 (84.8–96.6)	100, 77/77 (95.3–100)	6	0
	VersaTREK	95.6, 87/91 (89.2–98.3)	94.7, 54/57 (85.6–98.2)	4	3
*vanA*	BacT/Alert	100, 4/4 (51.0–100)	87.5, 28/32 (71.9–95.0)	0	4
	Bactec	100, 1/1 (20.7–100)	100, 5/5 (56.6–100)	0	0
	VersaTREK	93.3, 14/15 (70.2–98.8)	97.1, 33/34 (85.1–99.5)	1	1
*vanB*	BacT/Alert	100, 1/1 (20.7–100)	97.1, 33/34 (85.1–99.5)	0	1
	Bactec	NA, 0/0 (NA)	100, 6/6 (60.1–100)	0	0
	VersaTREK	100, 1/1 (20.7–100)	100, 47/47 (92.4–100)	0	0

a NA, not applicable.

b FN, false negative; FP, false positive.

A total of seven specimens were identified as mixed cultures for iC-GPC Assay target organisms by one or both assays (Table 3). One discrepant mixed sample was identified by the iC-GPC Assay to contain a target not detected by the Verigene BC-GP assay. The false-positive *vanA* result was confirmed by PCR/bidirectional sequencing to be negative for *vanA*. One discrepant mixed sample was identified by the Verigene BC-GP assay to contain targets not detected by the iC-GPC Assay. The false-negative S. aureus and *mecA* targets were confirmed as true positives by PCR/bidirectional sequencing.

**TABLE 3:** Comparative performances of mixed-culture specimens

iC-GPC Assay identification	Verigene BC-GP identification	Discrepant analysis results^*a*^
S. aureus, E. faecalis	S. aureus, E. faecalis	NA
S. epidermidis, *mecA*, E. faecalis	S. epidermidis, *mecA*, E. faecalis	NA
E. faecalis, E. faecium, *vanA*	E. faecalis, E. faecium	*vanA* negative
S. aureus, *mecA*, E. faecalis, *vanB*	S. aureus, *mecA*, E. faecalis, *vanB*	NA
S. epidermidis, *mecA*, E. faecalis	S. epidermidis, *mecA*, E. faecalis	NA
E. faecium, *vanA*	S. aureus, *mecA*, E. faecium, *vanA*	S. aureus, *mecA* positive
S. aureus, E. faecalis	S. aureus, E. faecalis	NA

a NA, not applicable.

Overall, the iC-GPC Assay compared favorably to the Verigene BC-GP assay, with an overall percent agreement of 95.5%. After arbitration testing of all discordant results between the iC-GPC Assay and Verigene BC-GP assay, the iC-GPC Assay agreed with the PCR/sequencing method for 31 specimens, while the Verigene method had agreement for 36 specimens. These results demonstrate that the iC-GPC Assay performs comparably to the Verigene BC-GP assay in this study.

## DISCUSSION

Gram-positive cocci are important causes of bloodstream infections, of which S. aureus, S. epidermidis, S. pneumoniae, E. faecalis, and E. faecium represent the most common isolates. Rapid characterization of these bacteria, along with the detection of their major antibiotic resistance determinants, favorably impacts patient care and outcomes. Patient survival rates directly correlate with the early characterization of these isolates, as mortality rates can increase 7.6% for every hour that treatment is delayed (20).

As is common with most molecular assays intended for BSI diagnosis, the iC-GPC Assay detects the most common resistance markers for methicillin and vancomycin resistance. Rapid detection of *mecA*, *vanA*, and *vanB* can inform treatment decisions days before traditional antimicrobial susceptibility testing (AST) results are available. However, the resistance markers detected by the iC-GPC Assay are not the only mechanisms of resistance to their respective antibiotic classes. For example, genes, such as *mecB*, *mecC*, and an altered penicillin-binding 4 (PBP4) gene, have all been shown to confer resistance to methicillin and other beta-lactams in Staphylococcus spp. (21). Additionally, Enterococcus spp. can contain genes, like *vanC* and *vanD*, that confer glycopeptide resistance apart from the more common *vanA* and *vanB* mechanisms (22). In general, bacteria are constantly evolving mechanisms to combat antibiotics. As such, no PCR-based molecular diagnostic will be able to detect all genes that confer resistance to a specific class of antibiotics, let alone single nucleotide polymorphisms. For these reasons, all molecular diagnostics recommend performing traditional culture and AST analysis in parallel in case undetected mechanisms of antibiotic resistance are present in an isolate.

The results of this study have shown that the iC-GPC Assay is comparable in performance to the Verigene BC-GP assay and provides a reliable molecular alternative for the detection of five of the most common causes of Gram-positive bacterial bloodstream infections and their antibiotic resistance determinants. As with all molecular assays, there are drawbacks to the iC-GPC Assay. First, the iC-GPC Assay has limited species coverage. While the assay does not cover all the causative organisms of Gram-positive bloodstream infections, it does cover the most common and clinically relevant species. Many of the species not covered in the assay are considered common blood culture contaminants. Off-panel organisms, such as Micrococcus spp., can be pathogenic but are more commonly detected as contaminants (19, 23, 24). The limitations of a smaller panel can also be seen in a positive light given that the cost per test will be lower than those for some of the more expansive panels currently on the market. A second limitation of the assay is the 4-h run time. The iC-GPC Assay has a longer run time than several other assays currently on the market. However, the open-access nature of the iC-Processor and the flexible time to testing minimize the impact of the longer run time.

While drawbacks exist for the iC-GPC Assay and for molecular diagnostics in general, the iCubate system offers several benefits. First, the iC-GPC Assay is performed in a single closed-system disposable cassette. This feature allows for a very simplified “load and go” assay that requires only a few minutes of a technologist's setup time. Loading the cassette is quick and easy, requiring only a single pipetting step, and it involves no additional dilutions, reagents, hydrations, or tubes. Second, the use of the closed-system cassette greatly reduces the risk of aerosolized infectious microorganisms during specimen processing and amplicon contamination. This not only protects lab personnel, but it also essentially eliminates the chance of carryover contamination between assays. Third, the iC-Processor is capable of simultaneous random access processing of up to four cassettes on an instrument platform that has a relatively small footprint (17″ by 17″ by 16″). If a laboratory requires greater throughput, additional iC-Processors can be linked together while only requiring a single iC-Reader. Finally, the iC-GPC Assay provides a significantly more affordable alternative, particularly for small- to medium-sized health care institutions, for the molecular detection of common Gram-positive bacteria responsible for bloodstream infections and their resistance markers. Collectively, these benefits may positively impact technologist safety, workflow, costs, and throughput compared to other FDA-cleared molecular platforms and direct detection blood culture assays. Most importantly, the use of direct detection molecular blood culture assays, such as the iC-GPC Assay, will significantly reduce morbidity and mortality rates, shorten hospital stays, decrease costs, and favorably impact hospital antibiotic stewardship programs (25–29).
